# Development of a Copro-RPA-CRISPR/Cas12a assay to detect *Echinococcus granulosus* nucleic acids isolated from canine feces using NaOH-based DNA extraction method

**DOI:** 10.1371/journal.pntd.0012753

**Published:** 2024-12-12

**Authors:** Guoqing Shao, Xiaowei Zhu, Ruiqi Hua, Yanxin Chen, Guangyou Yang

**Affiliations:** Department of Parasitology, College of Veterinary Medicine, Sichuan Agricultural University, Chengdu, P. R. China; James Cook University, AUSTRALIA

## Abstract

**Background:**

Cystic echinococcosis (CE), caused by *Echinococcus granulosus* sensu lato (*E*. *granulosus* s.l.), remains a significant zoonotic parasitic disease affecting both livestock and humans. It arises from the ingestion of food and water contaminated with canine feces containing *E*. *granulosus* eggs. The detection of these eggs in canine feces is essential for guiding effective preventative measures against the disease. Therefore, the development of a novel accurate, rapid, and visually interpretable point-of-care test is crucial for controlling CE.

**Methods:**

We combined recombinase polymerase amplification (RPA) and Clustered Regularly Interspaced Short Palindromic Repeats (CRISPR) with a CRISPR-associated protein 12a (Cas12a) system, forming the RPA-CRISPR/Cas12a assay. This assay targeted the *E*. *granulosus* mitochondrial *nad2* gene and utilized a lateral flow strip for visual readout. To improve field applicability, we integrated a simple and cost-effective NaOH-Based DNA extraction method. Clinical validation included testing DNA extracted from eighteen canine fecal samples, followed by comparison with quantitative PCR (qPCR) and two commercial enzyme-linked immunosorbent assay (ELISA) kits.

**Results:**

The RPA-CRISPR/Cas12a assay showed a detection limit of 1 fg/μL DNA, without any cross-reactivity with related tapeworms such as *Echinococcus multilocularis*, *Dipylidium caninum*, *Taenia hydatigera*, *Taenia multiceps*, and *Taenia pisiformis*. When applied to 62 clinical fecal samples from dogs, the RPA-CRISPR/Cas12a assay demonstrated 68% sensitivity, while the developed RPA-CRISPR/Cas12a-NaOH assay exhibited 45% sensitivity. In the field performance comparison of the RPA-CRISPR/Cas12a and the RPA-CRISPR/Cas12a-NaOH assay with qPCR and two ELISA kits, the sensitivity, consistency rate, and Youden’s index suggested good or fair agreement with the currently employed detection methods.

**Conclusion:**

This study describes the development and validation of the RPA-CRISPR/Cas12a and RPA-CRISPR/Cas12a-NaOH assays for detecting *E*. *granulosus* in canine feces. The developed assays surpassed previous detection methods in providing enhanced diagnostic sensitivity and enabling point-of-care testing. Moreover, these assays hold potential for surveilling *E*. *granulosus* in low-income countries.

## Author summary

Cystic echinococcosis (CE) is a neglected zoonotic parasitic disease with global health significance. This impactful parasitic disease, resulting from the ingestion of food and water contaminated with canine feces containing *E*. *granulosus* eggs, poses a threat to both livestock and humans. Detection of canine feces contaminated with *E*. *granulosus* eggs will guide effective preventative measures for local governments, especially in low-income countries. Various tools have been developed to detect *E*. *granulosus*, among which ELISA kits are currently the most used in *E*. *granulosus* prevention and control. However, these tests face challenges related to low sensitivity and high cross-reactivity. Conversely, nucleic acid detection methods offer high specificity and stability, but often require skilled technicians and expensive infrastructure, limiting their applicability in low-income countries lacking such resources. To address these limitations, we introduce a novel assay with promising potential for field application, integrating recombinase polymerase amplification (RPA) and Clustered Regularly Interspaced Short Palindromic Repeats (CRISPR) alongside a CRISPR-associated protein 12a (Cas12a) system (RPA-CRISPR/Cas12a). For enhancing field utility, we innovatively propose a straightforward and cost-effective NaOH-Based DNA extraction method integrated into this assay. The newly devised RPA-CRISPR/Cas12a assay outperforms previous detection tests, offering improved diagnostic sensitivity and enabling point-of-care detection. This diagnostic technique holds promise as a potential point-of-care testing method for nucleic acid detection of *E*. *granulosus*, especially in canine feces contaminated with parasite eggs, and is expected to play a crucial role in securing the health of livestock and humans in low-income countries.

## Introduction

*Echinococcus granulosus* sensu lato (s.l.) is a tapeworm that resides in the intestines of canines [1]. The eggs of *E*. *granulosus* s.l. can be excreted in canine feces, posing a risk of ingestion by livestock or humans, ultimately causing a significant parasitic disease known as cystic echinococcosis (CE) [2]. Cystic echinococcosis continues to be a threat to human and animal health, causing substantial economic losses [3]. Recent epidemiological investigations have underscored *E*. *granulosus* as the prevailing genotype within *E*. *granulosus* s.l., being responsible for more than 90% of infections [4–6]. Effectively managing CE poses a significant challenge. Dogs serve as the definitive host for *E*. *granulosus*, thus playing a pivotal role in CE transmission [7,8]. The dog population is significantly smaller than that of intermediate hosts; therefore, the elimination of infected dogs has emerged as a feasible and practical strategy for CE control [8,9]. Consequently, the establishment of an accurate and straightforward detection method is imperative to identify *E*. *granulosus* infections in dogs.

Various tools have been developed over recent decades to detect *E*. *granulosus*. The gold standard for *E*. *granulosus* diagnosis in dogs is post-mortem inspection [10]. However, this method relies on skilled technicians and necessitates costly infrastructure. Immunological diagnostic methods to detect the canine coproantigen of *E*. *granulosus* have also been introduced. Despite their suitability for large-scale diagnosis and commercial availability, coproantigen tests still present challenges related to low sensitivity and high cross-reactivity [11–13]. Conversely, numerous DNA-based detection methods, such as copro-DNA-PCR and loop-mediated isothermal amplification (LAMP), have been tailored for field applications [14–16]. Presently, PCR-based detection methods are predominantly carried out in laboratories because of their requirements for technicians and devices. Additionally, LAMP, as an isothermal amplification method, exhibits heightened detection sensitivity and minimal device requirements, with significant potential for field surveillance [17]. Despite the advantages of LAMP, such as its high sensitivity, its stringent operational protocols necessitate trained personnel with a controlled experiment environment to prevent false-positive results [18].

In recent years, innovative DNA detection methods based on Clustered Regularly Interspaced Short Palindromic Repeats (CRISPR)-associated protein 12a (Cas12a) have emerged, demonstrating notable advantages in specificity, sensitivity, and operational convenience for field applications [19]. Cas12a, when guided by a predesigned CRISPR RNA (crRNA), exhibits the ability to selectively target double-stranded DNA (dsDNA) or single-stranded DNA (ssDNA). Following the targeting of DNA sequences, the unique trans-cleavage activity of Cas12a is activated, leading to the nonspecific degradation of the ssDNA detection reporter in the reaction system [20]. To enhance the sensitivity of the Cas12a system, the integration of recombinase polymerase amplification (RPA), an isothermal amplification technique, is commonly employed [21]. The successful amalgamation of RPA and CRISPR/Cas12a detection has been demonstrated in the diagnosis of various pathogens, including SARS-CoV-2 [22], *Staphylococcus aureus* [23], and *Toxoplasma gondii* [24]. Additionally, CRISPR/Cas12a assays have been successfully utilized for detecting a range of parasites, including several helminths and protozoans [25]. However, to the best of our knowledge, no such method has been developed to detect *E*. *granulosus*.

Moreover, DNA extraction using commercial kits is prohibitively expensive and impractical for field applications. Consequently, there is a demand for a cost-effective NaOH-based DNA extraction method that can be seamlessly integrated with the detection system. Previous studies have demonstrated the robust tolerance of RPA to inhibitors present in alkaline-lysed blood samples [26]. In this pilot investigation, we aimed to establish an innovative detection system for *E*. *granulosus* by employing NaOH-based DNA extraction combined with the RPA reaction with the Cas12a detection system. Furthermore, for the visual interpretation of the results, a lateral flow strip readout was incorporated. The developed detection system for *E*. *granulosus*, characterized by rapidity, specificity, sensitivity, and convenience, holds significant potential for use as a point-of-care test (POCT) in low-income areas.

## Materials and methods

### Ethics statement

The animal study was reviewed and approved by the Animal Care and Use Committee of Sichuan Agricultural University (SYXK2019-187). All animal procedures used in this study were carried out in accordance with the Guide for the Care and Use of Laboratory Animals (National Research Council, Bethesda, MD, USA) and recommendations of the ARRIVE guidelines (https://www.nc3rs.org.uk/arrive-guidelines). All methods were carried out in accordance with relevant guidelines and regulations.

### Detection kits and methods

In this study, a comparative analysis was conducted between RPA-CRISPR/Cas12a or RPA-CRISPR/Cas12a-NaOH and two enzyme-linked immunosorbent assay (ELISA) kits, along with a quantitative polymerase chain reaction (qPCR) method. Table 1 presents detailed information about the employed kits. Notably, the ELISA kits under comparison are currently used in the prevention and control of *E*. *granulosus* in China.

**Table 1:** Major features of the assessed tests for *E*. *granulosus* diagnosis in canine feces.

Tests	Description	Time required	Manufacturer
ELISA	Currently marketed and used in China	90 min	Shenzhen Combined Biotech Co. Ltd (Lot Number:20220927)
Sandwich ELISA	Currently marketed and used in China	130 min	Beijing Tian Tech Co. Ltd (Lot Number:EG-ES2305)
qPCR	SYBR Green quantitative PCR	120 min	This study
RPA-CRISPR/Cas12a	RPA/CRISPR/Cas12a assay	105 min	This study
RPA-CRISPR/Cas12a-NaOH	RPA/CRISPR/Cas12a assay using a NaOH-Based DNA extraction method	55 min	This study

### Collection of samples and DNA extraction

Sixty-two positive canine fecal samples were collected from two distinct sources, as detailed in Table 2: six laboratory-reared dogs at two different time points, and fifty dogs from endemic areas in Ganzi or Aba, Sichuan province, China. After these dogs were euthanized, positive cases were identified by demonstrating the presence of worms in the intestine, recognized as the definitive "gold" standard to identify *E*. *granulosus* infections [27]. Subsequently, *E*. *granulosus* was detected in euthanized dogs, confirming the infection, with recorded parasite burdens ranging from 712 to 8,760 worms.

**Table 2:** Composition and origin of the canine fecal samples.

Category	Number of dogs sampled	Sample code	Sample origin
Clinical canine fecal samples	Twelve positive canine feces were collected from six artificially infected laboratory dogs at 21days post-infection (dpi) and 28 dpi, respectively. Twenty-six positive and twenty-four negative samples collected from dogs in endemic areas were approved by the Sichuan Center for Animal Disease Prevention and Control.	Six samples from artificially infected laboratory dogs at 21 dpi were coded as number 1,2,3,4,5,6; those at 28dpi were coded as 7,8,9,10,11,12. Twenty-six samples from infected dogs in endemic areas were coded as 13–38, while negative samples were coded as 39–62.	Sichuan Agricultural University; Sichuan Center for Animal Disease Prevention and Control.
Negative canine fecal samples	Five negative canine fecal samples were from five laboratory-reared dogs.	These samples were coded as N1-N5.	Sichuan Agricultural University.
Artificial positive canine fecal samples	500 mg of feces from a parasite-free dog spiked with 5, 10, 20, 40, and 80 *E*. *granulosus* s. s. PSCs were used as positive controls.	These samples were coded as P5, P10, P20, P40 and P80.	Sichuan Agricultural University.

To assess the detection limit of the RPA-CRISPR/Cas12a-NaOH method, five negative canine fecal samples were obtained from laboratory-reared dogs without any parasitic infection. Positive controls were prepared by spiking 500 mg of feces from a parasite-free dog with 5, 10, 20, 40, and 80 protoscoleces (PSCs). Before detection, all fecal samples were stored at -80°C for one week to mitigate biosafety risks.

For specificity evaluation, seven additional samples were included: *Canis lupus familiaris* genomic DNA, *Escherichia coli* genomic DNA (both prevalent in canine feces), and genomic DNA from related tapeworms: *Echinococcus multilocularis*, *Dipylidium caninum*, *Taenia hydatigena*, *Taenia multiceps*, and *Taenia pisiformi*.

The DNA from all samples was extracted using a DNA Stool Mini Kit (Tiangen, Beijing, China), following the provided instructions.

### Primer, crRNA, and probe design

The mitochondrial genomes of *E*. *granulosus* and other relevant *Taenia* species were retrieved from the National Center for Biotechnology Information (NCBI) database. Subsequently, MEGA7 software was employed with default settings to align all the sequences. Primer and crRNA design utilized Primer Premier 5 (Premier Biosoft, Palo Alto, CA, USA) and CRISPR-offinder software [28], respectively. In silico specificity testing of the primers was conducted using NCBI BLAST and primer-BLAST (NCBI, Bethesda, MD, USA). Synthesis of all primers, CRISPR RNA, and a single-stranded DNA reporter (ssDNA-FQ) was carried out by Sangon Biotech (Shanghai, China) (Table 3).

**Table 3:** Primers, CrRNA, and the probe used in this study.

Name		Sequence (5’-3’)
RPA primers	RPA-F	GTGTATCGTGTATTTAGAGTTGGTAGATGGGTG
	RPA-R	GTAGAAACAGACGATAACGAAATATGACA
qPCR primers	q-F	TATTTAGAGTTGGTAGATGGGTG
	q-R	GTAGAAACAGACGATAACG
CRISPR RNA	Cr-RNA	UAAUUUCUACUAAGUGUAGAUAUACAAAAAACAGAAAAAUA
ssDNA reporter	Reporter	6-FAM-TTTTTATTATATTTT-Biotin

### The RPA reaction

The RPA procedure adhered to the guidelines provided by the RPA basic kit (Amp-future Co., Ltd, Shandong, China). The reaction was conducted at 37°C for 20 minutes in a total volume of 50 μL, comprising 2 μL of forward primer (10 μM), 2 μL of reverse primer (10 μM), 29.4 μL of Buffer A, 2.5 μL of Buffer B, 10.1 μL of ddH₂O, and 4 μL of DNA template.

### The RPA-CRISPR/Cas12a combined with a lateral flow strip detection assay

The optimal reaction time of the Tiosbio Cas 12/13 Dedicated Nucleic Acid Test Strips (JY0301, Tiosbio, Beijing, China) was determined following the provided instructions at room temperature for various times: 0, 5, and 10 min; 1 h, and more than 2 h.

Subsequently, the CRISPR/Cas12a assay was conducted at 37°C for 20 minutes in a 20 μL volume, comprising 1 μL of LbaCas12a (1 μM) (New England Biolabs, Ipswich, MA, USA), 1 μL of crRNA (2 μM), 0.25 μL of RNase inhibitor (New England Biolabs), 0.5 μL of ssDNA reporter (10 μM) (Sangon Biotech, Shanghai, China), 2 μL of 10 × NEBuffer 2.1 (New England Biolabs), 13.25 μL of H₂O, and 2 μL of the RPA product. Following CRISPR/Cas12a detection, ddH₂O was added to the reaction mixture to reach a total volume of 100 μL, and the mixture was applied to the lateral flow strip. Results were obtained at 5 min and recorded using a cellphone camera.

### Specificity evaluation of RPA-CRISPR/Cas12a

The specificity of the RPA primer set was assessed within the RPA-CRISPR/Cas12a assay using DNA obtained from various samples. This evaluation involved testing the specificity of the RPA-CRISPR/Cas12a assay against genomic DNA extracted from *Canis lupus familiaris* intestinal tissue and *E*. *coli*. These DNA sources were chosen because of their abundance in canine feces. Additionally, the assay’s specificity was scrutinized against genomic DNA derived from tapeworms commonly found in canine feces, namely: *E*. *multilocularis*, *D*. *caninum*, *T*. *hydatigena*, *T*. *multiceps*, and *T*. *pisiformis*.

### Limit of detection (LOD) of the RPA-CRISPR/Cas12a assay

For the LOD test, specific segments of the *E*. *granulosus* mitochondrial *nad2* gene were amplified using RPA primers (Table 3) and subsequently cloned into pMD-19T plasmid (Takara, Dalian, China). These plasmids were then introduced into the *E*. *coli* DH5a strain (Tiangen) and extracted using a Plasmid Extraction Kit (Tiangen). The resulting recombinant plasmid underwent serial dilution (1:10) ranging from 10 ng to 1 fg and then used to assess the LOD of the RPA-CRISPR/Cas12a assay.

### Pilot study of the RPA-CRISPR/Cas12a combined with an NaOH-Based DNA extraction method

To initiate the NaOH-based DNA extraction, 500 mg of each fecal sample was suspended in 1 mL of phosphate-buffered saline (PBS) and vortexed for 15 seconds. Subsequently, the suspensions were mixed with 0.2 M NaOH (1:1) and incubated at 95°C for 10 minutes. Afterwards, the mixtures were briefly centrifuged (6,000× g, 3 min), and the upper liquid phase was diluted with Tris-HCl (0.2 M) at ratios of 1:1, 1:10, 1:20, and 1:40. Finally, 4 μL of each dilution served as a template for detection.

**Fig 1:**
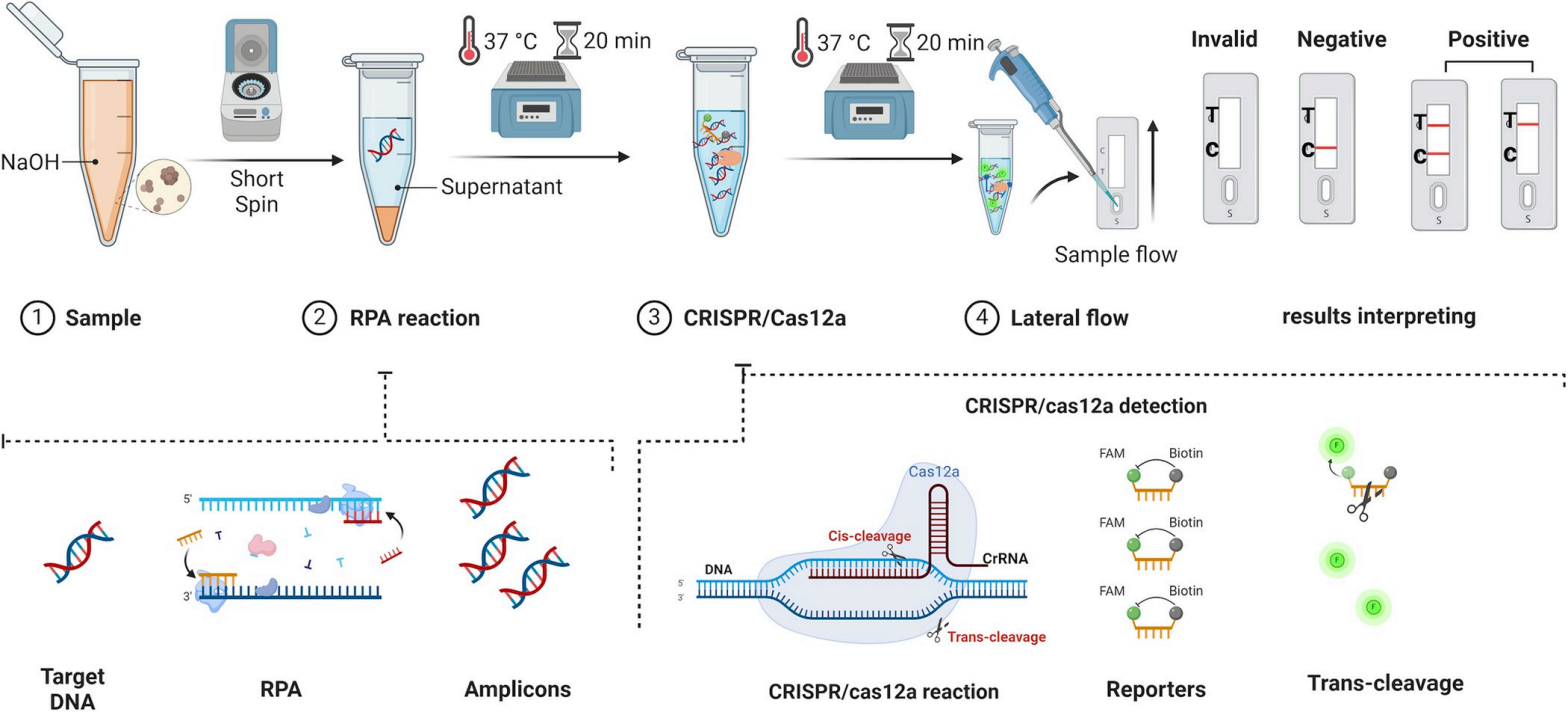
The workflow (Created with BioRender.com) of the RPA-CRISPR/Cas12a-NaOH assay. After the canine feces were processed with NaOH, the supernatant was used for the RPA reaction (37°C for 20 min). Then, the CRISPR/Cas12a reaction system was used to bind to the DNA targets, which triggered the collateral activity of Cas12a, resulting in cleavage of the ssDNA reporters. Finally, the reporters were visualized using a lateral flow test strip.

The RPA-CRISPR/Cas12a-NaOH procedure was executed following the schematic representation depicted in Fig 1. After the NaOH-Based DNA Extraction of canine feces, the RPA reaction was carried out at 37°C for 20 minutes in a total volume of 50 μL, comprising 2 μL of forward primer (10 μM), 2 μL of reverse primer (10 μM), 29.4 μL of Buffer A, 2.5 μL of Buffer B, 10.1 μL of ddH₂O, and 4 μL of the sample post-treatment, as detailed in Section 2.8.1. Subsequently, the CRISPR/Cas12a assay was executed as previously described.

The LOD to detect *E*. *granulosus* using the RPA-CRISPR/Cas12a-NaOH assay was assessed through serial dilutions of artificial positive canine fecal samples. These samples were serially spiked with 5, 10, 20, 40, and 80 PSCs, serving as positive controls. Meanwhile, five negative canine fecal samples from laboratory-reared dogs were utilized as negative controls. Each of the canine fecal samples underwent duplicate testing.

### SYBR Green quantitative PCR detection and standard curve

The SYBR Green qPCR detection targeting the *nad2* gene was executed using the LightCycler System (Roche, Basel, Switzerland). The procedure comprised a 20 μL reaction volume consisting of 10 μL of 2 × TB Green Premix Ex Taq II (Takara, Shiga, Japan), 1 μL of primer F, 1 μL of primer R, 2 μL of DNA template, and 6 μL of ddH₂O. The amplification conditions were set as follows: an initial denaturation at 95°C for 2 min; 40 cycles of denaturation at 95°C for 10 seconds, and annealing and amplification at 58°C for 30 seconds; followed by a final cooling step at 16°C. To establish the standard curve for quantification, a pMD-19T plasmid (Takara, Dalian, China) containing the *nad2* gene was 10-fold diluted ranging from 10 ng/μL to 1 fg/μL was employed.

### Application of RPA-CRISPR/Cas12a, RPA-CRISPR/Cas12a-NaOH, ELISA, Sandwich ELISA, and qPCR in field samples

Each of the canine fecal samples outlined in Table 2 underwent duplicate testing with each detection method. The ELISA and Sandwich ELISA procedures were conducted following the manufacturer’s instructions, while the remaining detection methods were implemented in accordance with the protocols described in this study.

### Data analysis

Data analysis was conducted using the SPSS 20.0 software package (IBM, Armonk, USA). The key indicators considered for analysis included sensitivity, consistency rate, and the Youden’s index.

Sensitivity: This metric represents the proportion of known infected fecal samples that tested positive in an assay, where infected fecal samples that tested negative were considered false negatives. Sensitivity is calculated using the formula: Sensitivity = TP / (TP + FN) × 100% (TP, true positive; FN, false negative).

Specificity: The proportion of uninfected reference fecal samples that tested negative in the assay (with uninfected fecal samples testing positive regarded as false positives) was calculated. The formula for the specificity: Specificity = TN/ (FP + TN) × 100% (TN, true negative; FP, false positive).

Consistency Rate: This denotes the proportion of samples with the same test results as the actual results of the reagents. The formula for consistency rate: Consistency Rate = (TP + TN) / N × 100% (TN, true negative; N: total number of samples).

Youden’s index: Youden’s index measures the overall effectiveness of an assay in detecting both true positive and true negative samples. The formula for Youden’s index: Youden’s index = (Sensitivity + Specificity)-1.

## Results

### Primer and CRISPR RNA design

The mitochondrial *nad2* gene was chosen as the target sequence for primers and crRNA design because of its conserved sequence in *E*. *granulosus* and varied sequences among other species. Multiple sequence alignment analysis, based on BLAST results using the targeted fragment, confirmed the suitability of the primers and crRNA (S1 Fig).

### The lateral flow strip reaction time

**Fig 2:**
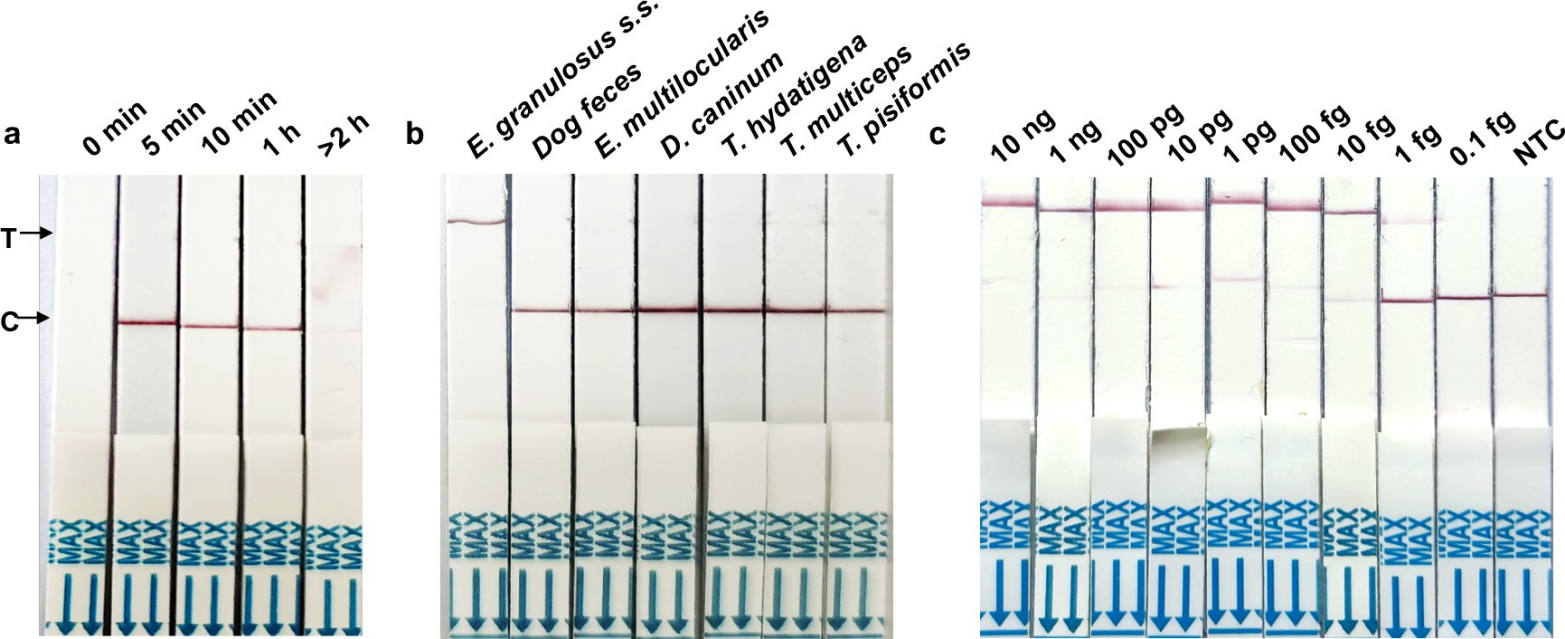
Determination of the detection performance of the RPA-CRISPR/Cas12a assay. (a) The lateral flow strip’s reaction time was optimized using several time settings for the RPA-CRISPR/Cas12a assay. Determination of the specificity (b) and sensitivity (c) of the RPA-CRISPR/Cas12a assay.

The assessment of the lateral flow strip’s reaction time involved testing a wide range of time settings. The findings revealed that the strip could yield results within 5 mins at room temperature. However, if the reaction time was beyond 2 h, the results were sometimes deemed invalid (Fig 2A). To achieve a shorter overall reaction time, we selected 5 minutes as the duration for the lateral flow strip reaction.

### Analytical specificity of the RPA-CRISPR/Cas12a assay

The RPA primers aimed were designed to introduce variability from other prevalent tapeworm species inhabiting the canine intestine. The NCBI Blast results demonstrated the absence of cross-reactivity with other parasites or host genomes. In our specificity test, the primer set successfully identified *E*. *granulosus* as positive, as evident from a distinct band at the test line on the flow strip. No other parasites tested positive using these primers (Fig 2B).

### Analysis of the LOD of the RPA-CRISPR/Cas12a assay

To assess the sensitivity of the RPA-CRISPR/Cas12a assay to detect the *E*. *granulosus* mitochondrial *nad2* gene, the pMD-19T*-nad2* plasmid was serially diluted from 10 ng to 1 fg per reaction. Fig 2C illustrates that the LOD for the RPA/CRISPR/Cas12a assay was determined as 1 fg, corresponding to the detection limit observed in qPCR within this study. Negative results were recorded when the test band was weaker than that of the 1 fg sample, which had been pretreated with the DNA extraction kit.

### Development and LOD of the RPA-CRISPR/Cas12a-NaOH assay

**Fig 3:**
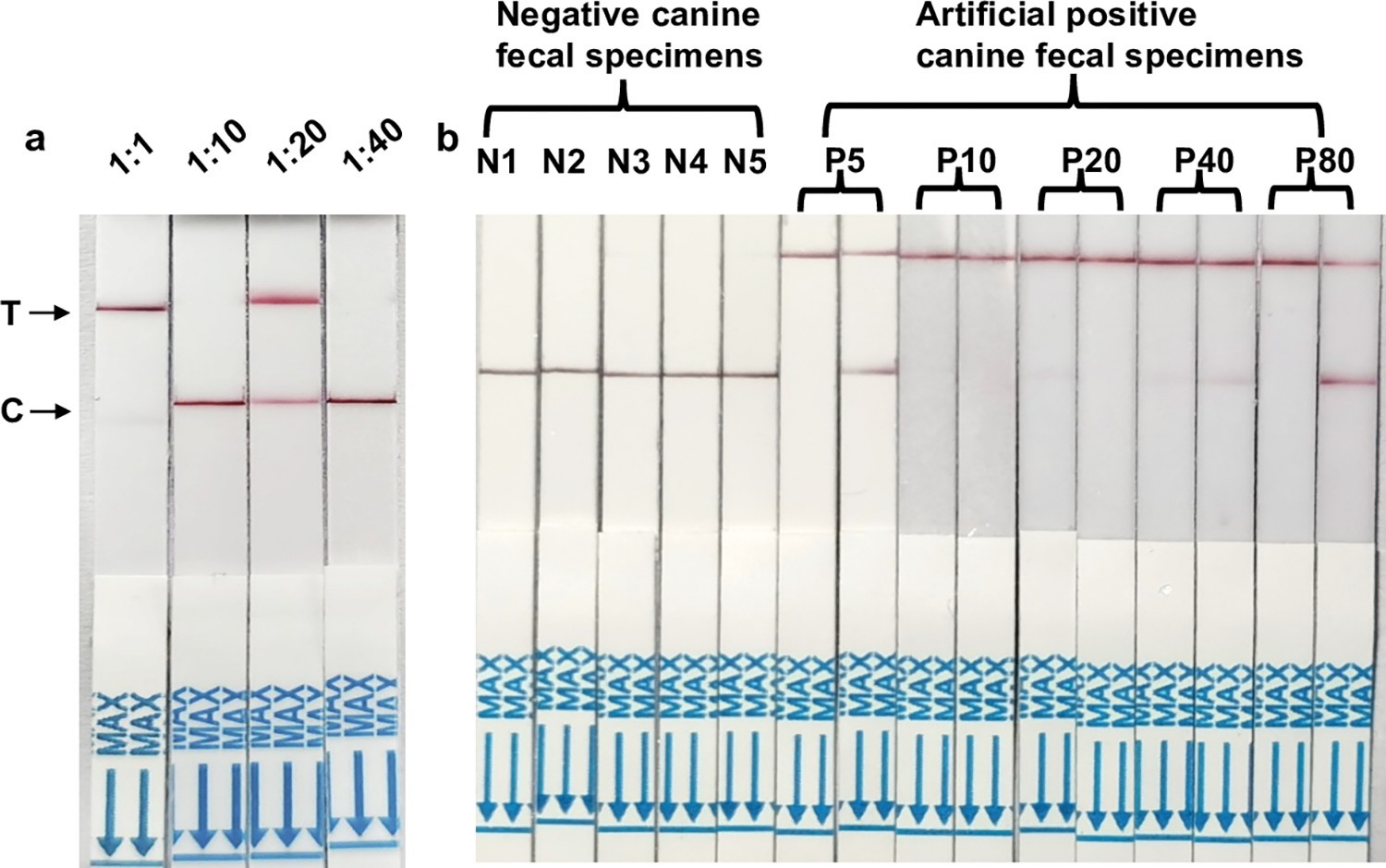
Determination of the detection performance of the RPA-CRISPR/Cas12a-NaOH assay. (a) To optimize the combination of the RPA-CRISPR/Cas12a and NaOH-based DNA extraction reaction system, we used extracted DNA at four different dilutions. (b) Determination of the sensitivity of the RPA-CRISPR/Cas12a-NaOH assay with negative canine feces N1-N5 and canine feces mixed with 5 (P5), 10 (P10), 20 (P20), 40 (P40) and 80 (P80) PSCs, respectively.

We successfully integrated the NaOH-based DNA extraction method to enhance and broaden the applicability of this test for general field use. The sample suspensions were combined with 0.2 M NaOH in a 1:1 ratio and incubated at 95°C for 10 minutes. Following centrifugation, the supernatant was mixed with Tris-HCl (0.2 M) in a 1:1 ratio (Fig 3A). When we tested the sample at a 1:1 dilution, positive results were consistently reproducible across experiments. However, samples diluted to 1:10, 1:20, or 1:40 tested with the assay yielded unstable results. Therefore, we concluded that a 1:1 dilution was optimal. Finally, 4 μL of each dilution served as a template for the RPA-CRISPR/Cas12a reaction, as described. The LOD for the RPA-CRISPR/Cas12a-NaOH assay was determined to be five PSCs in artificial positive canine fecal specimens (Fig 3B). Negative results were recorded when the test band was identical to that of the negative samples, which had been pretreated using the NaOH-based DNA extraction method.

### SYBR Green real-time PCR detection, standard curve, and LOD

**Fig 4:**
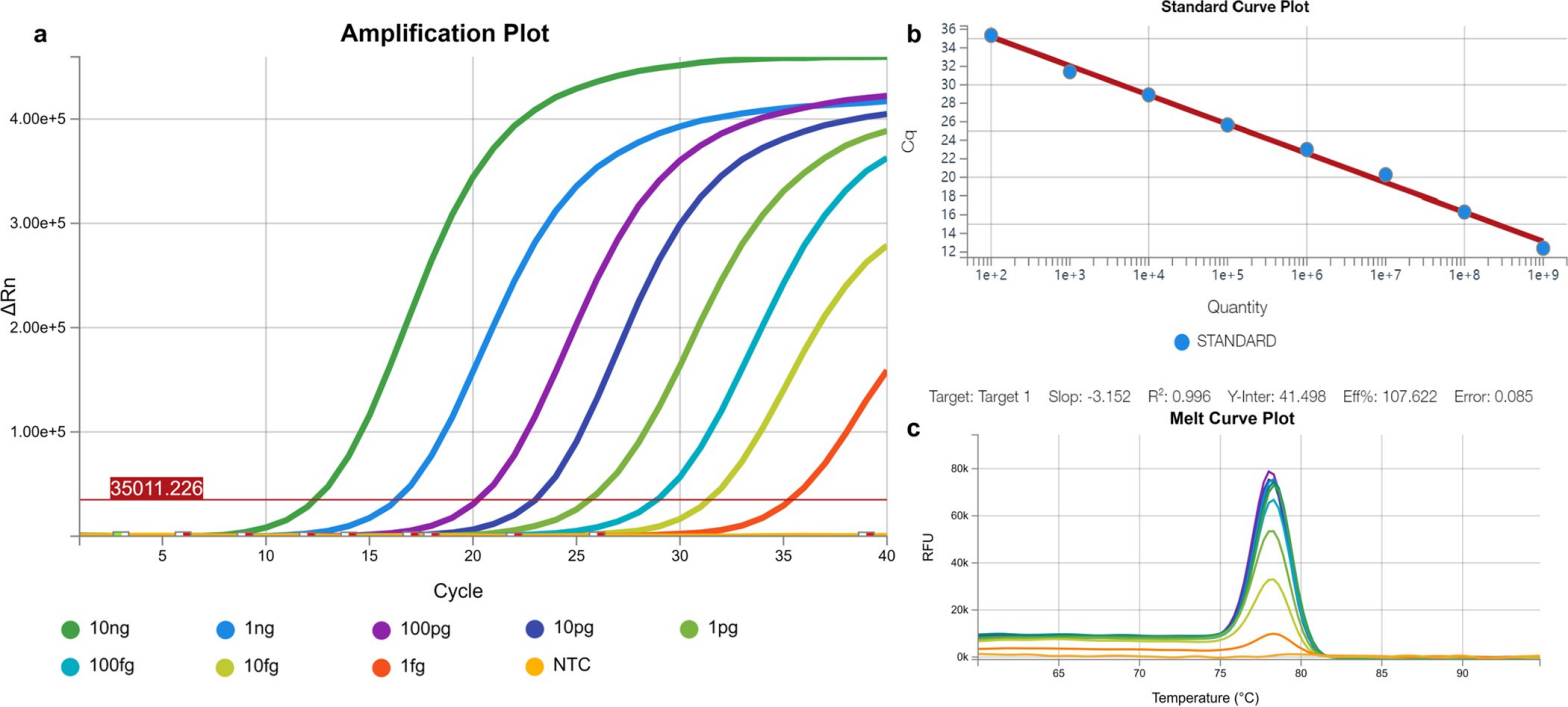
The qPCR amplification plot of the *E*. *granulosus* pMD-19T-*nad2* plasmid serially diluted from 10 ng to 1 fg per reaction (a) and standard curves generated from quantification cycle (Cq) values (b). Melting curves for *E*. *granulosus* pMD-19T-*nad2* plasmid showed one peak, and the melting temperatures were between 75 and 80°C (c).

We established and assessed the standard curve, revealing a highly significant relationship value (R^2^ = 0.996), with a slope within the accepted criterion (-3.152) (Fig 4). The LOD for qPCR was determined as 1 fg, aligning with the detection limit observed in the RPA-CRISPR/Cas12a method in this study.

### Field performance the RPA-CRISPR/Cas12a and RPA-CRISPR/Cas12a-NaOH assays compared with ELISA, Sandwich ELISA, and qPCR assays

**Fig 5:**
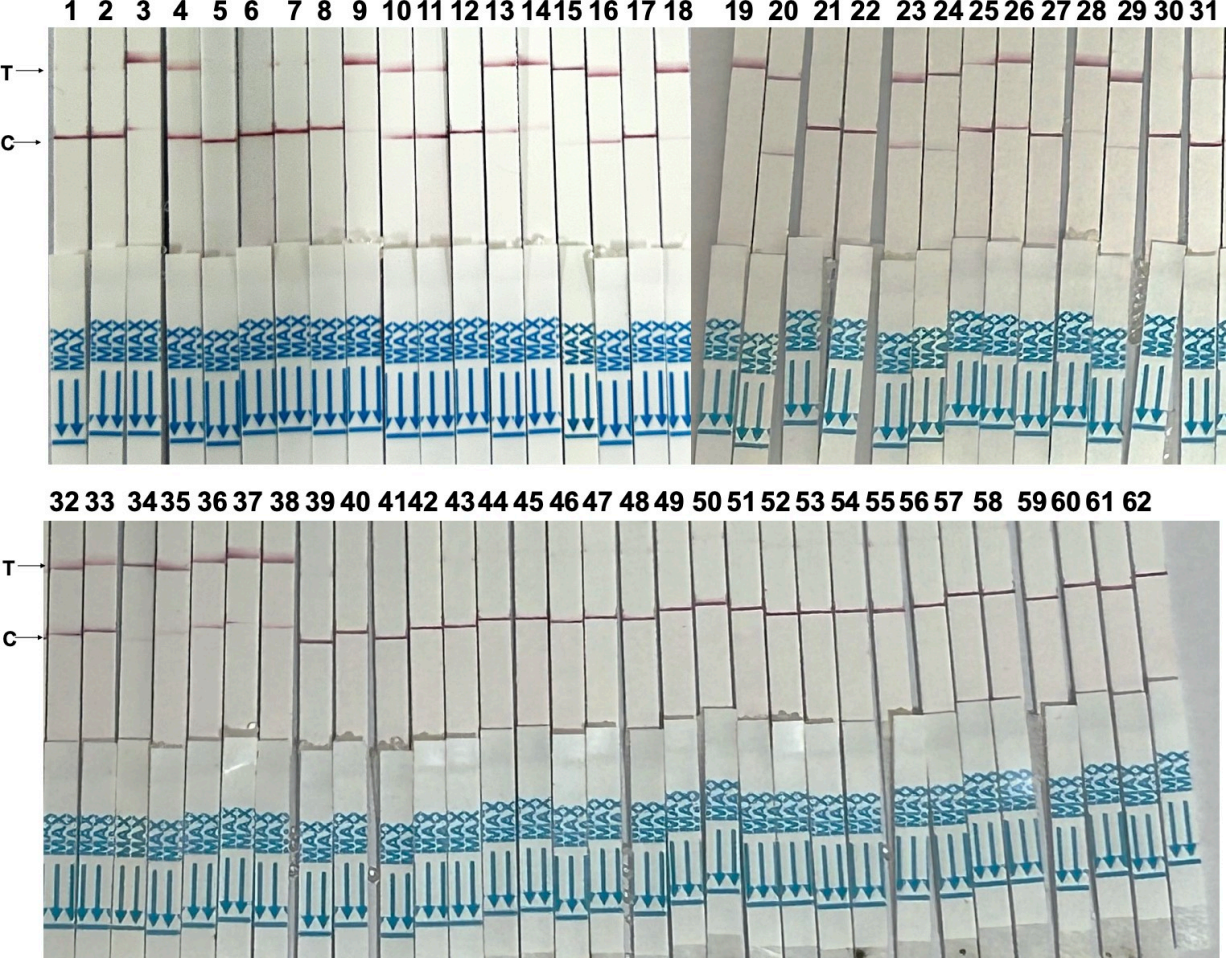
Results of the RPA-CRISPR/Cas12a assay to detect 62 canine fecal samples.

For all 62 clinical fecal samples collected from dogs (S1 Table), the RPA-CRISPR/Cas12a assay demonstrated higher sensitivity (68%), specificity (100%), consistency rate (81%), and Youden’s index (68%) in detecting *E*. *granulosus* in canine feces compared to indirect ELISA and Sandwich ELISA, while showing comparable results to qPCR (Fig 5 and Table 4).

**Table 4:** The field performance of qPCR, ELISA, Sandwich EILSA, RPA-CRISPR/Cas12a and RPA-CRISPR/Cas12a-NaOH to detect *E*. *granulosus* in 62 clinical canine feces.

Tests	N	TP	TN	FP	FN	Youden’s index	Consistency rate	Specificity	Sensitivity
qPCR	62	26	24	0	12	68%	81%	100%	68%
ELISA	62	9	22	2	29	15%	50%	92%	24%
Sandwich EILSA	62	20	21	3	18	40%	66%	88%	53%
RPA-CRISPR/Cas12a	62	26	24	0	12	68%	81%	100%	68%
RPA-CRISPR/Cas12a-NaOH	62	17	24	0	21	45%	66%	100%	45%

**Fig 6:**
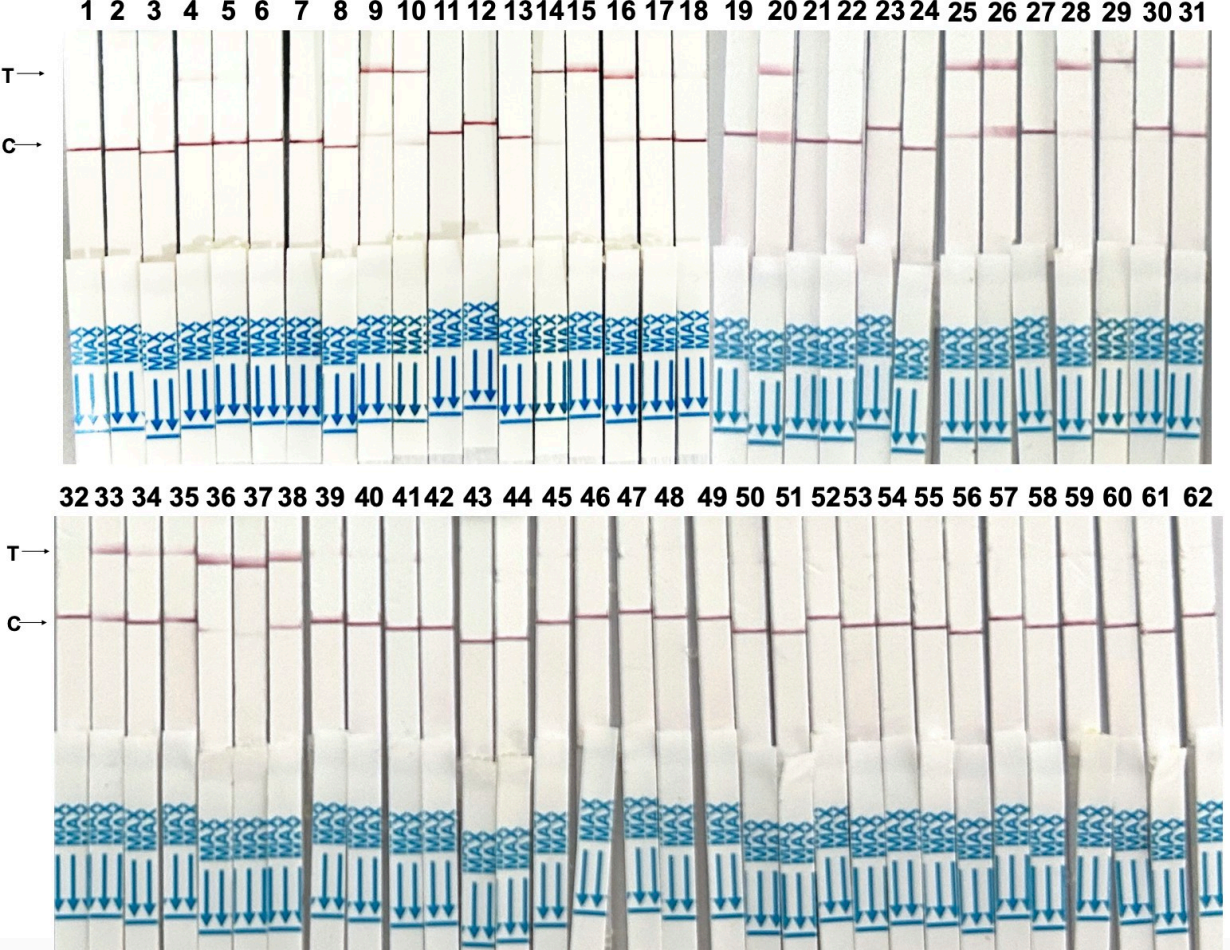
Results of the RPA-CRISPR/Cas12a-NaOH assay to detect 62 canine fecal samples.

In the field performance comparison of the RPA-CRISPR/Cas12a-NaOH assay with ELISA, Sandwich ELISA, and qPCR, the Youden’s index (45%) and specificity (100%) of the RPA-CRISPR/Cas12a-NaOH assay were superior to those of ELISA kits. However, its sensitivity (45%) was lower than that of Sandwich ELISA (53%), with both assays showing the same consistency rate (66%) (Fig 6 and Table 4).

For the fifty clinical fecal samples collected from dogs in endemic areas, the RPA-CRISPR/Cas12a assay demonstrated equal sensitivity to qPCR (81%) and higher sensitivity than both ELISA (27%) and Sandwich ELISA (50%) in detecting *E*. *granulosus* in canine feces. Furthermore, the RPA-CRISPR/Cas12a assay showed the highest Youden’s index (81%), matching that of qPCR (Table 5).

**Table 5:** The field performance of qPCR, ELISA, Sandwich EILSA, RPA-CRISPR/Cas12a and RPA-CRISPR/Cas12a-NaOH to detect *E*. *granulosus* in 50 clinical fecal samples from dogs in endemic areas.

Tests	N	TP	TN	FP	FN	Youden’s index	Consistency rate	Specificity	Sensitivity
qPCR	50	21	24	0	5	81%	90%	100%	81%
ELISA	50	7	22	2	19	19%	58%	92%	27%
Sandwich EILSA	50	13	21	3	13	38%	68%	88%	50%
RPA-CRISPR/Cas12a	50	21	24	0	5	81%	90%	100%	81%
RPA-CRISPR/Cas12a-NaOH	50	15	24	0	11	58%	78%	100%	58%

Meanwhile, the RPA-CRISPR/Cas12a-NaOH assay also exhibited enhanced sensitivity (58%) and Youden’s index (58%) in these samples, outperforming both ELISA and Sandwich ELISA across all evaluated metrics (Table 5).

## Discussion

Cystic echinococcosis persists as a neglected parasitic zoonosis with global health significance, as underscored by the World Health Organization (WHO) roadmap for 2021–2030 [29]. *E*. *granulosus* infection is pivotal in the context of CE, affecting both livestock and humans [1]. Epidemiological investigations consistently highlight the correlation between CE infections and poverty, particularly emphasizing the deficient management of stray dogs [4,30,31]. Given that the precise field diagnosis of infected dogs is crucial to control CE transmission in livestock and humans [32,33], it is imperative to devise a straightforward, accurate, and cost-effective POCT diagnostic method. This is especially important to detect *E*. *granulosus* infections in dogs in low-income regions.

In underdeveloped regions, local governments face challenges in allocating resources for the professional staff and expensive equipment required for to *E*. *granulosus* surveillance, impeding the efficiency of controlling CE. In response to this challenge, we devised an innovative, rapid, and cost-effective diagnostic approach for *E*. *granulosus* using the RPA-CRISPR/Cas12a assay. Integrating RPA with CRISPR/Cas12a and employing a lateral flow readout makes the method adaptable for field tests, with straightforward operation and results interpretation [34]. The RPA-CRISPR/Cas12a assay has been used as a nucleic acid detection method for various pathogens, including parasites [24,35].

In this study, the RPA-CRISPR/Cas12a assay for detecting *E*. *granulosus* demonstrated acceptable sensitivity (68%) and perfect specificity (100%). Notably, when applied to clinical canine feces from endemic areas, the assay exhibited performance comparable to qPCR and demonstrated superior sensitivity (81%) compared to the two ELISA methods evaluated. This increased sensitivity may be due to the presence of *E*. *granulosus* eggs in the samples, which likely provided sufficient detectable DNA [15]. In contrast, the sandwich ELISA failed to detect several samples that were identified as positive through nucleic acid detection, possibly because of the greater stability of DNA detection methods compared with ELISAs. Additionally, the Youden’s index of the RPA-CRISPR/Cas12a assay demonstrated excellent agreement with those of the qPCR method, particularly in the detection of clinical samples. Moreover, the sensitivity of the RPA-CRISPR/Cas12a assay surpassed that of the other nucleic acid detection methods [14,36], providing outstanding performance to detect *E*. *granulosus* in canine feces.

In this study, the sensitivities of the RPA-CRISPR/Cas12a assay and qPCR were comparable; however, their associated costs differ. The cost per test is approximately $5 for qPCR, whereas the RPA/CRISPR/Cas12a assay costs $2 for RPA and $3 for the lateral flow strip [37]. However, qPCR entails additional costs for equipment and technician, making it a potentially more expensive option. Furthermore, qPCR relies on a PCR polymerase that is sensitive to reaction inhibitors, necessitating complex DNA purification processes with associated costs ranging from $3 to $11 per sample for commercial DNA extraction kits. In contrast, the RPA/CRISPR/Cas12a method potentially leverages the use of alkaline-lysed samples, reducing the reaction time to less than 1 hour, a notable improvement over the time-consuming process of PCR. Unlike the PCR method, the RPA-CRISPR/Cas12a does not depend on costly equipment; all reactions can be conducted at 37°C, and the results can be interpreted visually using the lateral flow strip. Consequently, the RPA-CRISPR/Cas12a assay offers distinct advantages for field application.

Existing protocols for the RPA-CRISPR/Cas12a assay often require nucleic acid extraction using commercial DNA extraction kits [19,21]. In pursuit of enhanced field convenience, our study innovatively integrated the NaOH-Based DNA extraction method with the RPA-CRISPR/Cas12a assay. The outcomes revealed robust tolerance of the RPA-CRISPR/Cas12a assay’s reaction system to chemical inhibitors [38]. Previous refinements of the RPA-CRISPR/Cas12a assay primarily focused on optimizing the reaction system and results presentation, whereas our study indicated the potential for further enhancement through a simplified nucleic acid extraction method. Building on the success of the RPA-CRISPR/Cas12a assay, we developed and evaluated the performance of the RPA-CRISPR/Cas12a-NaOH detection method in a pilot study. This method demonstrated lower performance compared to the RPA-CRISPR/Cas12a assay when a simple NaOH-Based DNA extraction method was used. Chemical inhibitors (such as proteinases, bile salts, polyphenols, and acids) in the reaction system partially affected detection efficacy. Nonetheless, it exceeded ELISA methods in sensitivity (58%), specificity (100%), consistency rate (78%), and Youden’s index (58%) for detecting *E*. *granulosus* in fifty clinical fecal samples from dogs in endemic areas. However, sensitivity dropped to 45% when tested on sixty-two samples, including twelve from dogs with prepatent infections, indicating that the RPA-CRISPR/Cas12a assay had limited effectiveness in detecting prepatent infections. Meanwhile, its LOD is five PSCs; thus, if the clinical samples contain several eggs, this might provide sufficient DNA to exploit the superior LOD performance of the RPA-CRISPR/Cas12a-NaOH assay. The main transmission route of CE to livestock and humans is through canine feces contaminated with *E*. *granulosus* eggs [1], and this method is effective to screen dog fecal samples with biosafety concerns, ensuring public health through a simple POCT assay.

There are certain limitations in our study that warrant acknowledgment. Firstly, although the use of a lateral flow strip for visual result readout is convenient, weak positive bands are difficult to recognize, often requiring retesting or confirmation with other methods. Secondly, the RPA-CRISPR/Cas12a-NaOH process involved three steps for completion, which might represent too many operational steps, potentially restricting its field application. Our future endeavors will focus on optimizing the reaction conditions to streamline the process to two steps [39].

## Conclusions

Our study provides support for the efficacy of the RPA-CRISPR/Cas12a or RPA-CRISPR/Cas12a-NaOH assay to detect *E*. *granulosus* in canine feces, demonstrated by their satisfactory LOD, specificity, and sensitivity. Moreover, the innovative RPA-CRISPR/Cas12a-NaOH assay shows promise as a POCT method for nucleic acid detection of *E*. *granulosus*, particularly in feces contaminated with worm eggs. This contamination remains a significant biosafety concern for both livestock and humans, especially in low-income regions.

## Supporting information

S1 Fig Sequence alignment analysis, based on BLAST results using the targeted fragment, showed the primers and crRNA, which were marked with arrows and symbols, respectively. (TIF)

S2 Fig Specificity determination of the qPCR assay. (TIF)

S1 Table The detailed data for qPCR, ELISA, Sandwich ELISA, RPA-CRISPR/Cas12a and RPA-CRISPR/Cas12a-NaOH assays to detect 62 canine fecal samples. (PDF)
