# Supplementary figures and images for: Development of a Copro-RPA-CRISPR/Cas12a assay to detect Echinococcus granulosus nucleic acids isolated from canine feces using NaOH-based DNA extraction method
Source: PLoS Negl Trop Dis. 2024 Dec 12;18(12):e0012753. doi: 10.1371/journal.pntd.0012753 (PMC11671004; doi:10.1371/journal.pntd.0012753)

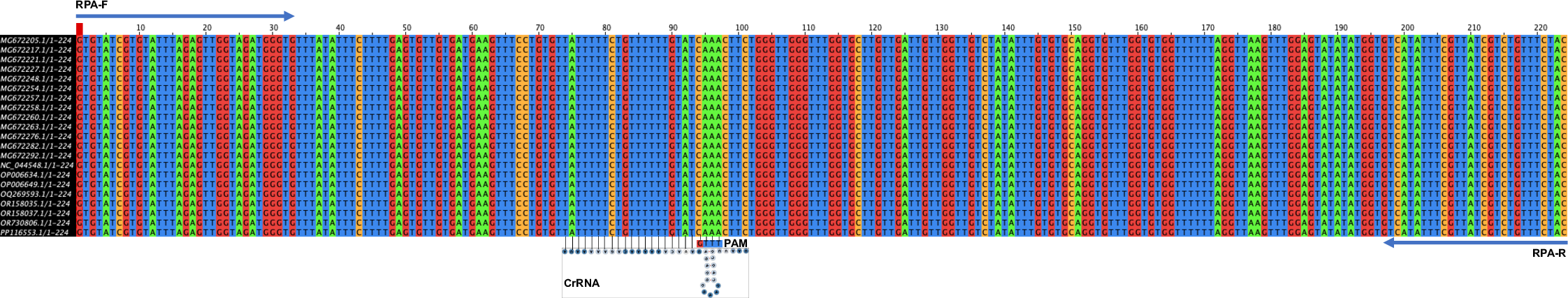

Supplement: S1 Fig — (TIF) [file pntd.0012753.s001.tif]

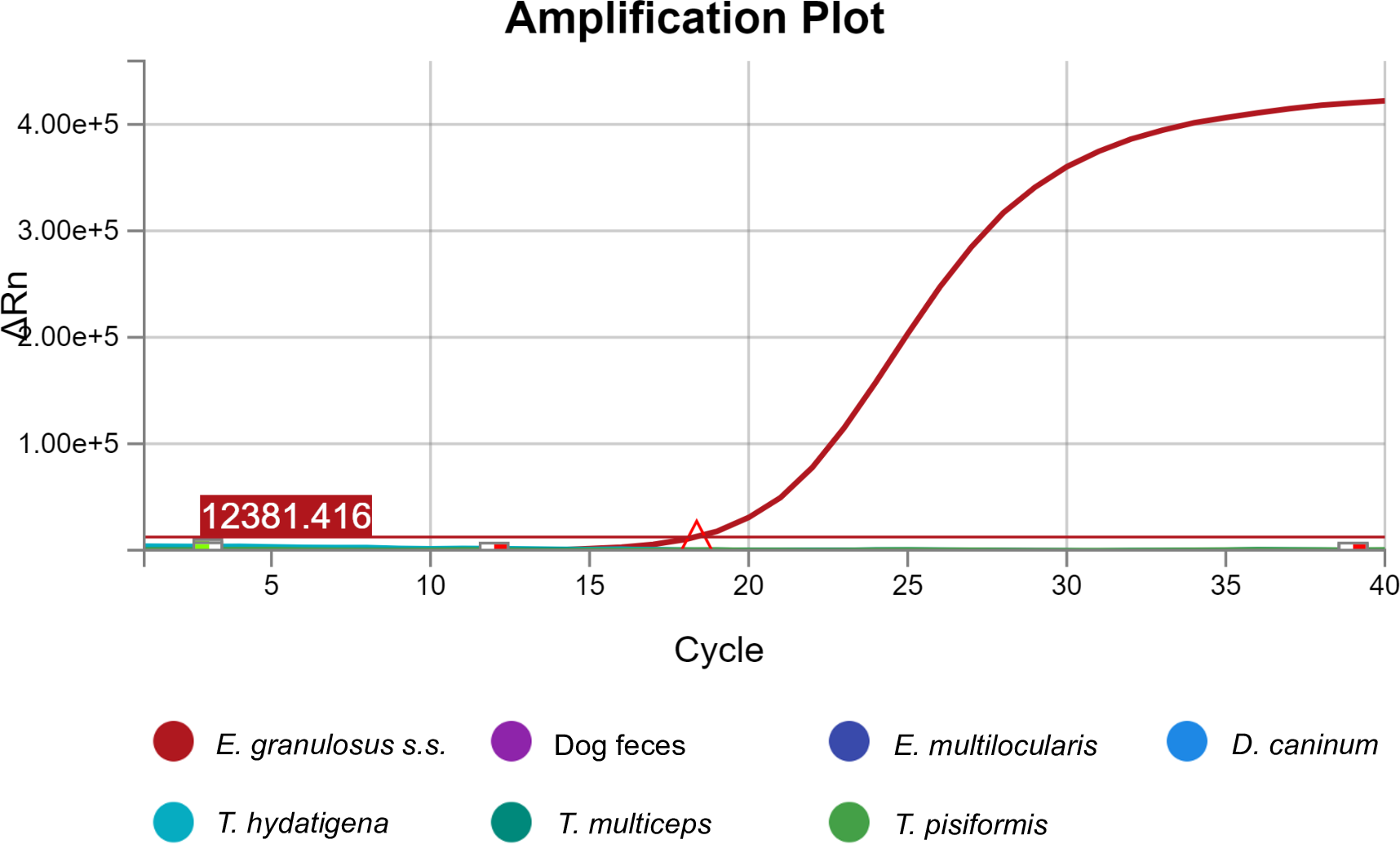

Supplement: S2 Fig — (TIF) [file pntd.0012753.s002.tif]
