# Supplementary material for: Development of a Copro-RPA-CRISPR/Cas12a assay to detect Echinococcus granulosus nucleic acids isolated from canine feces using NaOH-based DNA extraction method
Source: PLoS Negl Trop Dis. 2024 Dec 12;18(12):e0012753. doi: 10.1371/journal.pntd.0012753 (PMC11671004; doi:10.1371/journal.pntd.0012753)
Supplement: S1 Table — (PDF) [file pntd.0012753.s003.pdf]

Table S1. Results of qPCR, ELISA, Sandwich ELISA, RPA-CRISPR/Cas12a and RPA-CRISPR/Cas12a-NaOH assays to detect 62 canine fecal specimens.

Sample code	Sample note	qPCR (Ct value)	ELISA (OD450;Cut-off value=0.315)	Sandwich ELISA (OD450;Cut-off value=0.229)	RPA-CRISPR/Cas12a	RPA-CRISPR/Cas12a-NaOH
1	Pre-patent infection (21 dpi)	/	0.17	0.09	Negative	Negative
2	Pre-patent infection (21 dpi)	/	0.12	0.17	Negative	Negative
3	Pre-patent infection (21 dpi)	30.55	0.12	0.62	Positive	Negative
4	Pre-patent infection (21 dpi)	32.52	0.42	0.62	Positive	Negative
5	Pre-patent infection (21 dpi)	/	0.15	1.22	Negative	Negative
6	Pre-patent infection (21 dpi)	/	0.13	0.16	Negative	Negative
7	Pre-patent infection (28 dpi)	/	0.15	0.35	Negative	Negative
8	Pre-patent infection (28 dpi)	/	0.16	0.33	Negative	Negative
9	Pre-patent infection (28 dpi)	26.92	0.18	0.62	Positive	Positive
10	Pre-patent infection (28 dpi)	31.54	0.62	1.56	Positive	Positive
11	Pre-patent infection (28 dpi)	25.96	0.16	0.18	Positive	Negative
12	Pre-patent infection (28 dpi)	/	0.14	0.45	Negative	Negative
13	Pre-patent or patent infection	31.01	0.52	0.65	Positive	Negative
14	Pre-patent or patent infection	32.18	0.51	0.09	Positive	Positive
15	Pre-patent or patent infection	26.93	1.11	0.40	Positive	Positive
16	Pre-patent or patent infection	29.48	0.11	0.08	Positive	Positive
17	Pre-patent or patent infection	/	0.24	0.08	Negative	Negative
18	Pre-patent or patent infection	32.40	0.19	0.09	Positive	Negative
19	Pre-patent or patent infection	32.10	0.18	0.09	Positive	Negative
20	Pre-patent or patent infection	30.41	0.16	0.25	Positive	Positive
21	Pre-patent or patent infection	/	0.20	0.08	Negative	Negative
22	Pre-patent or patent infection	/	0.15	0.09	Negative	Negative
23	Pre-patent or patent infection	30.05	0.18	0.08	Positive	Negative
24	Pre-patent or patent infection	32.57	0.17	0.09	Positive	Negative
25	Pre-patent or patent infection	30.59	0.14	0.33	Positive	Positive
26	Pre-patent or patent infection	26.68	0.58	0.61	Positive	Positive
27	Pre-patent or patent infection	/	0.14	0.10	Negative	Negative
28	Pre-patent or patent infection	28.01	0.12	0.37	Positive	Positive
29	Pre-patent or patent infection	25.65	0.20	0.27	Positive	Positive
30	Pre-patent or patent infection	/	0.55	0.61	Negative	Negative
31	Pre-patent or patent infection	23.48	0.14	0.08	Positive	Positive
32	Pre-patent or patent infection	30.92	0.15	0.35	Positive	Negative
33	Pre-patent or patent infection	28.88	0.13	0.09	Positive	Positive
34	Pre-patent or patent infection	26.79	0.48	0.59	Positive	Positive
35	Pre-patent or patent infection	30.73	0.15	0.37	Positive	Positive
36	Pre-patent or patent infection	29.23	1.12	0.62	Positive	Positive
37	Pre-patent or patent infection	29.92	0.18	0.08	Positive	Positive
38	Pre-patent or patent infection	28.58	0.15	0.10	Positive	Positive
39	Negative	/	0.17	0.09	Negative	Negative
40	Negative	/	0.16	0.10	Negative	Negative
41	Negative	/	0.45	0.58	Negative	Negative
42	Negative	/	0.16	0.09	Negative	Negative
43	Negative	/	0.18	0.09	Negative	Negative
44	Negative	/	0.12	0.10	Negative	Negative
45	Negative	/	0.13	0.08	Negative	Negative
46	Negative	/	0.15	0.08	Negative	Negative
47	Negative	/	0.14	0.39	Negative	Negative
48	Negative	/	0.37	0.10	Negative	Negative
49	Negative	/	0.15	0.08	Negative	Negative
50	Negative	/	0.16	0.09	Negative	Negative
51	Negative	/	0.21	0.08	Negative	Negative
52	Negative	/	0.11	0.08	Negative	Negative
53	Negative	/	0.16	0.08	Negative	Negative
54	Negative	/	0.16	0.08	Negative	Negative
55	Negative	/	0.13	0.24	Negative	Negative
56	Negative	/	0.16	0.09	Negative	Negative
57	Negative	/	0.20	0.10	Negative	Negative
58	Negative	/	0.21	0.09	Negative	Negative
59	Negative	/	0.18	0.08	Negative	Negative
60	Negative	/	0.20	0.09	Negative	Negative
61	Negative	/	0.16	0.09	Negative	Negative
62	Negative	/	0.17	0.09	Negative	Negative
NTC		/				
